# Genome-Wide Identification of Transcriptional Start Sites in the Plant Pathogen *Pseudomonas syringae* pv. *tomato* str. DC3000

**DOI:** 10.1371/journal.pone.0029335

**Published:** 2011-12-28

**Authors:** Melanie J. Filiatrault, Paul V. Stodghill, Christopher R. Myers, Philip A. Bronstein, Bronwyn G. Butcher, Hanh Lam, George Grills, Peter Schweitzer, Wei Wang, David J. Schneider, Samuel W. Cartinhour

**Affiliations:** 1 lant-Microbe Interactions Research Unit, Robert W. Holley Center for Agriculture and Health, Agricultural Research Service, United States Department of Agriculture, Ithaca, New York, United States of America; 2 Department of Plant Pathology and Plant-Microbe Biology, Cornell University, Ithaca, New York, United States of America; 3 Life Sciences Core Laboratories Center, Cornell University, Ithaca, New York, United States of America; 4 Laboratory of Atomic and Solid State Physics, Cornell University, Ithaca, New York, United States of America; Niels Bohr Institute, Denmark

## Abstract

RNA-Seq has provided valuable insights into global gene expression in a wide variety of organisms. Using a modified RNA-Seq approach and Illumina's high-throughput sequencing technology, we globally identified 5′-ends of transcripts for the plant pathogen *Pseudomonas syringae* pv. *tomato* str. DC3000. A substantial fraction of 5′-ends obtained by this method were consistent with results obtained using global RNA-Seq and 5′RACE. As expected, many 5′-ends were positioned a short distance upstream of annotated genes. We also captured 5′-ends within intergenic regions, providing evidence for the expression of un-annotated genes and non-coding RNAs, and detected numerous examples of antisense transcription, suggesting additional levels of complexity in gene regulation in DC3000. Importantly, targeted searches for sequence patterns in the vicinity of 5′-ends revealed over 1200 putative promoters and other regulatory motifs, establishing a broad foundation for future investigations of regulation at the genomic and single gene levels.

## Introduction

*Pseudomonas syringae* pv. *tomato* strain DC3000 (DC3000) is a phytopathogen of tomato and *Arabidopsis* and is the focus of many molecular plant-microbe interaction studies. Sequencing and annotation of the DC3000 genome and its two plasmids was completed by The Institute for Genome Research (TIGR) in 2003 [1]. Since then a number of genomic studies have revealed important details regarding conservation and distribution of Type III effectors, potential virulence factors, and the phylogenetic scope of *P. syringae* [2]. Although genome sequencing has provided a wealth of knowledge, the primary genome sequence represents only the first stage in understanding complex cellular processes that are critical to the survival of plant pathogens, such as sensing and responding to environmental signals. Since these behaviors rely on the coordinated expression of genome content, it is necessary to examine the transcriptome, proteome, and metabolome in detail.

RNA-Seq has emerged as a high-throughput strategy to analyze bacterial transcriptomes on a global scale (see reviews: [3]–[8]). This deep-sequencing approach has uncovered complex transcriptional activity, provided high-throughput validation of gene predictions, and efficiently revealed regulatory non-coding RNAs (ncRNAs), transcriptional start sites (TSSs), and antisense transcription in a number of bacteria. RNA-Seq protocols have been developed to target the 5′ region of transcripts, allowing the identification of larger numbers of putative transcriptional start sites and aiding in defining operons [9]–[11]. In addition, these approaches have detected and confirmed antisense activity [12]. Recently, a more efficient version of this strategy was employed to evaluate the primary transcripts of several human pathogens, *C. trachomatis* [13], *C. pneumoniae* [14] and *H. pylori* [15] and the cyanobacteria, *Synechocystis* [16]. These modified protocols exploit an enzyme that preferentially digests processed transcripts, and discriminates between primary and processed transcripts, resulting in a data set enriched for transcriptional start sites.

RNA-Seq has also been used to evaluate the transcriptome of DC3000 on a global scale [17]. This study generated valuable data concerning gene expression and provided important information that has been used for reannotation of the DC3000 genome [17]. However, very few confirmed transcriptional start sites have been reported for this pathogen. Furthermore, promoter models are available for only two sigma factors, HrpL and PvdS [18], [19]. Because the identification of promoters is critical to understanding gene regulation, we devised a protocol using Illumina's high-throughput sequencing strategy to experimentally determine transcriptional start sites for the DC3000 genome. Inspection of the DNA sequences upstream of the 5′-ends regions allowed us to identify the most likely sigma factor regulating many of these transcripts, setting the stage for new insights concerning gene regulation and expression in *P. syringae*.

## Materials and Methods

### Bacterial strains/growth conditions

DC3000 was routinely cultured on King's B agar [20] at 30°C. For RNA isolation, bacteria were cultured as previously described [21]. Briefly, bacteria were grown in iron-limited MG medium (10 g/liter mannitol, 2 g/liter L-glutamic acid, 0.5 g/liter KH_2_PO_4_, 0.2 g/liter NaCl, 0.2 g/liter MgSO_4_; final pH of 7.0) at 25°C in a bioreactor system (Sixfors). Bacterial cultures were collected at late exponential phase (optical density at 600 nm [OD_600_] of 0.6).

### RNA extraction

Total RNA was prepared with RNeasy kit (Qiagen, Valencia, CA) following the manufacturer's instructions, using the optional on-column DNaseI digestion and with the exception that lysozyme was used at a concentration of 5 mg/ml. RNA was treated twice with DNase I (Ambion, Austin, TX) to remove residual DNA and then cleaned and concentrated using the MinElute kit (Qiagen). Integrity of the RNA was assessed using the Agilent Bioanalyzer (Cornell University Life Sciences Core Laboratory Center [CLC] Microarray Facility, Cornell University).

### 5′ RACE

Transcriptional start points were determined using version 2.0 of Invitrogen's system for rapid amplification of cDNA ends (5′RACE) as described in Filiatrault *et al.* [17].

### Depletion of processed RNAs

RNA was heated to 60°C for 2 min and placed on ice. ∼2.5 µg of the total RNA was treated with 1 µl (5 units) of Terminator 5′-phosphate-dependent exonuclease (Epicentre) in a 20 µl reaction for 1 hour at 30°C according to the manufacturer's instructions. Several reactions were performed to enable treatment of 10 µg of total RNA. After incubation, reactions were terminated with 1 µl 100 mM EDTA (pH 8.0). RNA was purified and concentrated using the MinElute kit (Qiagen).

### Construction of cDNA libraries for 5′ mapping

The RNA (untreated or treated with Terminator exonuclease) was treated with 10 U tobacco acid pyrophosphatase (TAP, Epicentre Biotechnologies) in a 50 µl reaction for 1 hr at 37°C. RNA was purified using the MinElute kit (Qiagen). The terms “E−” and “E+” are used throughout the manuscript to distinguish the samples treated with either TAP alone (E− = no exonuclease treatment) or digestion with Terminator exonuclease (E+) followed by TAP.

The 3′-ends were blocked by treatment with NaIO_4_ to prevent recircularization, as described by [22]. An RNA oligonucleotide, 5′-ACAUCCUAGUAC-3′ (IDT custom RNA oligo) was ligated overnight to the available 5′-monophosphate ends in a 20 µl reaction at 16°C. The reaction was cleaned using the MinElute kit (Qiagen) and eluted in 14 µl H_2_O. First strand synthesis was performed using Superscript II (Invitrogen) and a random primer that also contained the sequence for the 3′ Illumina adapter [5′-CAAGCAGAAGACGGCATACGANNNNNN-3′]. The following cycling conditions were used: 25°C for 10 min, 42°C for 50 min, 70°C for 15 min. The RNA template was degraded by incubation with 1 µl of RNase H (Invitrogen). cDNA was purified and primer-dimers were removed with the Zymo kit (Zymo Research). cDNA was amplified using Phusion DNA polymerase (NEB) and the following primers: (5′-AATGATACGGCGACCACCGACAGGTTCAGAGTTCTACAGTCCGACGATCACATCCTAGTAC-3′) and (5′-CAAGCAGAAGACGGCATACGA-3′). The following PCR protocol was used: 1 min at 95°C, 15 cycles of: 15 seconds at 95°C, 30 seconds at 60°C and 20 seconds at 72°C, then 1 cycle for 7 min at 72°C. The libraries were purified using the Zymo-clean-up kit. Libraries were eluted in 12 µl of H_2_O. Library quality was assessed using the Agilent Bioanalyzer. Sequencing was performed on the Illumina Genome Analyzer II by the Cornell DNA Sequencing Core Facility.

### Read mapping and profile generation

Two libraries were prepared from two biological replicates for each condition (E1+, E2+, E1− and E2−). Sequence data obtained from each of these samples was transformed into histograms (“sinister profiles”) as described previously [17] with several modifications as follows. After conversion to FASTA format, the 12 nucleotides derived from the RNA adapter were trimmed from the original 43 nt read, leaving 31 nts. Trimmed reads were aligned to the DC3000 main chromosome (NC_04578) using SOAPalign/soap2 version 2.20 [23]. Reads that aligned perfectly to a single location were retained and all others were discarded. The value of the sinister profile at each genomic position is defined as the number of trimmed reads whose 5′-ends uniquely map to that position. Since our protocol ensures that strand-specificity is retained, there is independent data for each strand. When required, individual profiles for each condition were combined into a single profile by adding the counts at corresponding genomic locations, yielding two profiles, E12+ and E12−. Profiles were visualized using Artemis as previously described [17]. Profiles for the main chromosome can be found in Supplemental Information (Datasets, S1, S2, S3, S4, S5, S6).

### Reproducibility

In order to measure the reproducibility between biological replicates, comparisons were made between (E1+ vs. E2+ and E1− vs. E2−). Positive and negative strands from each individual profile were stored end-to-end, so that each one could be represented as a single column vector. Next, for each comparison, locations at which both vector's values were zero were removed. Finally, the Pearson's uncentered correlation measure was calculated for the pair of reduced vectors. The uncentered measure was used because, for sinister profiles, it produces more conservative results than the centered measure.

### Position Classification

Using the Genbank genome annotation of the DC3000 chromosome (AE016853.gbk, dated 01-DEC-2010, md5sum fec9f99c8c632ef92a6bde9719c44c7e), a locally written script was used to assign each genomic position on the chromosome to one of the following classes,

ANNOTATED: falling within and on the coding strand of an annotated gene.
OPPOSITE: falling within but on the non-coding strand of an annotated gene.
INTERGENIC: not falling within an annotated gene on either strand.

A profile containing the classification of each genomic position is found in Dataset S7. The values in the classification profile have the following meaning: 0 = intergenic; 1 = opposite; 2 = annotated.

### Visualizing correlation using scatter plots

The colored scatter plots were generated using the three-way classification and the Python script “make-class-scatter.py”, found in Dataset S8. For each genomic position, the values from the two profiles being compared are used as x and y coordinates. Points are binned to produce 2D histograms with 10,000 total bins (100 bins along each axis). Because the dynamic range of profile values and bin counts is so large, profile values are transformed using the function log_10_(1+x) before binning, and bin counts are then transformed using the function log_10_(1+log_10_ (1+x)). The bin “borders” are the same from plot to plot and thus profiles with different profile value distributions are effectively normalized and graphed at exactly the same scale so that they can be compared directly.

The number of positions assigned to the intergenic, opposite, and annotated classifications are counted separately in each bin. To generate a colored plot, primary colors are assigned using red for “intergenic” ends, green for “opposite” ends, and blue for “annotated” positions, using an RGB color code (http://www.w3.org/TR/REC-html32). The magnitude of counts in each component determines brightness with small counts being represented with dark colors and large counts with bright colors. Black represents 1, the lowest possible bin count. In this scheme, magenta represents a mixture of intergenic and opposite positions, cyan a mixture of opposite and annotated positions, and yellow a mixture of intergenic and opposite positions. White represents a mixture of all three classes with no class dominating. Counts have been clipped at 50% of the maximum count to improve printability. This brightens bins with low counts and fully saturates bins containing counts that exceed the clipping limit. Plots generated from unclipped counts appear in Figure S1.

### The E12+Top data set

The scatter plot (described above) was used to guide selection of a subset of positions from the E12+ profile exhibiting (a) insensitivity to exonuclease digestion, and (b) relatively high count values. The final cutoff at bin 22 isolates 5′-ends with count numbers exceeding 118 and corresponds to positions with the 2500 highest count values. Because more than one profile index has a value equal to the cutoff, the resulting list of positions includes 2510 indexes. We refer to this as the E12+Top data set. The corresponding GFF file is found in Dataset S9.

### Comparisons with global RNA-Seq data

A ratio of downstream and upstream transcription levels found in global transcript data [17] was computed for the positions in the E12+Top as follows. First, a 20-nucleotide region surrounding each position in the data set was defined. The region consisted of two windows; the downstream window included the position and nine nucleotides downstream of it (10 nucleotides total), and the upstream window consisted of ten nucleotides upstream of the position. All 20 genomic coordinates in the region were then tested to determine whether the 32-mer starting at each coordinate was unique in the DC3000 genome. The regions surrounding 2482 of 2510 positions in E12+Top data set passed this test. Next, read counts from the sinister profile from the stranded RNA-Seq experiment described in [17] were totaled for the upstream and downstream windows. These totals were used to compute a transition ratio using the following formula,
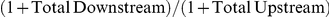
The geometric mean of the 2482 ratios is 3.61.

In order to evaluate the probability of a mean this large occurring by chance, the mean transition ratios for all positions across the DC3000 chromosome were computed. Then the geometric mean of 500,000 randomly chosen sets of 2482 positions were computed. None had a mean of 3.61 or greater (p-value<2e-6 (1/500,000)).

To determine whether the observed transitions were centered at the 5′-ends, a ratio was also computed using offsets of −10 to +10 surrounding the 5′-end positions in the global RNA-Seq data. A plot showing the average ratio over this range of offsets is shown in Figure S2.

### Promoter motif detection

E12+Top was used to generate a set of sequences that might contain promoter or other regulatory motifs by sampling 40 nucleotides upstream of each coordinate in the data set.

Abutting samples, and samples that overlapped by 1 or more nucleotides, were merged. The GFF file for the resulting sequence set is found in Dataset S10. The FASTA file for the 40 bp upstream sequences is in Supplementary Dataset S11. In order to find common sequence motifs, these sequences were used as input to MEME version 4.4.0 (http://meme.sdsc.edu/meme4_4_0/intro.html) with the following parameters,

-dna -mod anr -nmotifs 20 -minsites 2 -maxsites 2500
-minw 18 -maxw 40 -maxsize 200000

The first nine motifs generated (e-value<1) are presented in this report. The output from MEME is found in Dataset S12.

## Results

### Strategy for 5′-end capture, data generation, and filtering

**Figure 1:**
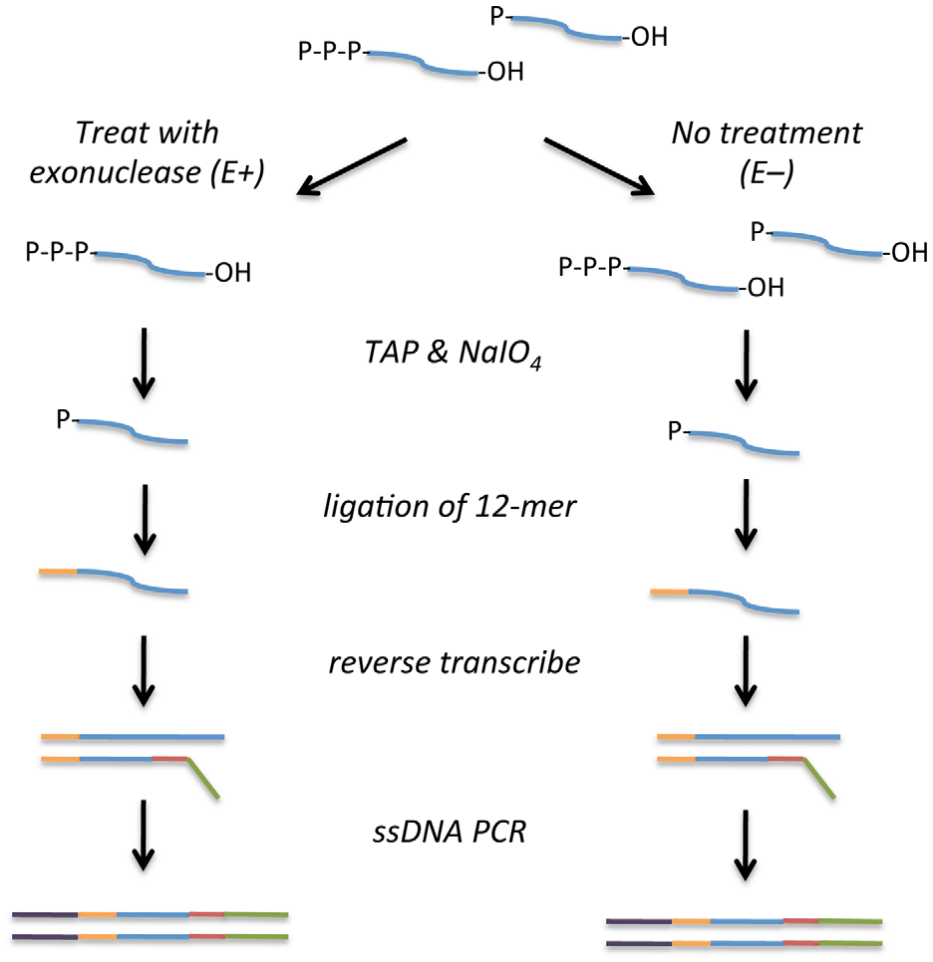
Strategy for 5′-end capture. Total RNA of *P. syringae* DC3000, with (E+) or without (E−) Terminator exonuclease treatment was treated with tobacco acid pyrophosphatase (TAP) to convert transcripts to 5′mono-phosphate RNA and sodium periodate to block 3′ hydroxyl groups. Next, a 12-nucleotide RNA linker was ligated to available 5′ends, and RNA was reverse transcribed using a random primer that contained the Illumina adapter sequence. Single stranded cDNA was PCR amplified using the Illumina adapter sequences.

The workflow for the experimental design is shown in Figure 1. RNA was isolated from *P. syringae* DC3000 grown in a bioreactor in MG medium under iron-limited conditions. The conditions and sampling schedule were identical to those used in our recent iron microarray [21] and the global RNA-seq [17] experiments. We prepared four libraries that consisted of two biological replicates treated with either TAP alone (E1− and E2−) or Terminator exonuclease followed by TAP (E1+ and E2+). Our strategy also incorporated a unique tag of 12 bases that was ligated to the available 5′-monophosphate to enable identification of the first base (the transcriptional start site). The rationale for incorporation of a tag was to ensure that we could unambiguously identify the transcriptional start site within the sequence read. RNA was reverse transcribed using a random primer that included the Illumina adapter used in the small RNA kit (Illumina, Inc.) followed by PCR to amplify only those products that contain both an adapter sequence and the 5′-end tag. Table 1 summarizes the reads and mapping statistics of the four libraries using data generated by the Illumina Genome Analyzer. Approximately 98% of the reads contained the 12 base 5′-tag, demonstrating that the approach had successfully captured 5′-ends. Because the vast majority of reads contain the tag, it is likely that the additional tagging was not necessary.

**Table 1:** Read mapping statistics.

Read type	E1−a	E2−	E1+	E2+
Total reads	15,504,125	10,975,486	8,479,640	8,100,502
Reads containing 12-mer	15,269,545	10,813,222	8,321,278	7,954,015
Reads that align uniquely to the chromosome	9,809,682	5,421,537	3,007,398	2,586,576

a Libraries were prepared from RNA that was either Treated with TAP alone (E−) or Terminator exonuclease and TAP (E+). 1 and 2 represent biological replicates.

**Figure 2:**
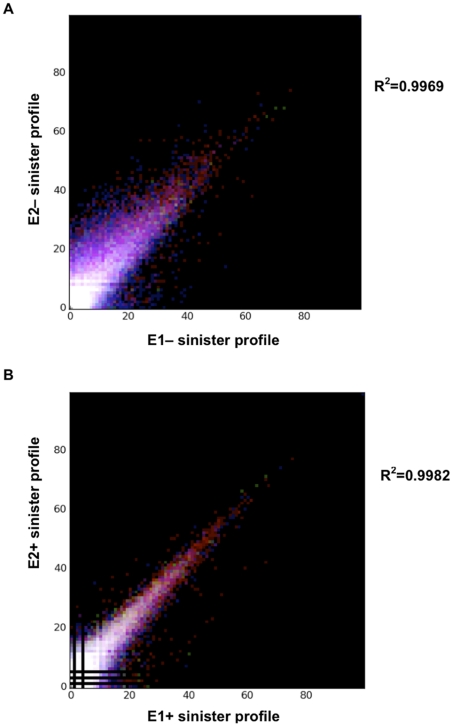
Correlation between biological replicates. In the correlation plots, each point indicates the reads counts. (A). Scatter plot of TAP-only treated biological replicate 1 (E1−) compared to TAP-only treated biological replicate 2(E2−). (B) Scatter plot of Exonuclease treated biological replicate 1 (E1+) compared to exonuclease treated biological replicate 2 (E2+). Correlation scores (R^2^) for each comparison are shown.

To assess reproducibility, biological replicates were compared (E1− vs. E2− and E1+ vs. E2+). To graphically illustrate the correlation, scatter plots of each pair of profiles were generated. Pearson's correlation coefficients of both comparisons, showed significant similarity (E1− vs. E2−, R^2^ = 0.9969; E1+ vs. E2+, R^2^ = 0.9982) between biological replicates (Figure 2), indicating high reproducibility between biological replicates from both conditions. Since the biological replicates displayed reproducibility, subsequent analyses were performed using merged data (E12− and E12+; see Methods). After combining the reads for each biological replicate, E12− and E12+ contained 15,231,219 and 5,593,974 reads, respectively. These reads correspond to 796,599 (E12−) and 429,492 (E12+) unique genomic locations in the DC3000 genome, representing 6.23% and 3.36% of possible locations (Table S1).

### Classification of 5′-ends

RNA 5′-ends may arise as a result of transcription initiation, processing, degradation, or other events. Treatment with Terminator exonuclease should enrich for sequence reads whose 5′-end is a transcriptional start site because the enzyme selectively degrades mRNAs bearing a monophosphate at their 5′-ends (if the end is exposed) [15], [16]. Primary transcripts, bearing 5′ triphosphates are resistant to treatment. We therefore expected that when the E12+ and E12− data sets are normalized and compared, 5′-ends susceptible to digestion by exonuclease will exhibit reduced numbers of sequence reads in E12+. In contrast, the reads for resistant 5′-ends should be present in similar numbers in the two data sets. The genomic distribution of resistant 5′-ends should also reflect an association with promoters. We therefore expect such 5′-ends to localize preferentially in intergenic regions where promoters are typically found.

In order to examine these predictions more closely, a classification scheme was devised to place 5′-ends in one of three exclusive categories based on their location. Briefly, the “annotated” category includes signals that lie within an annotated gene (i.e., on the annotated strand). The “opposite” category includes signals that fall into the non-coding strand of an annotated region. Finally, the remaining signals were classified as “intergenic”. The genomic distribution of captured 5′-ends varies among these classes depending on different cutoff values for the number of 5′-ends captured per position (see supplementary Table S2).

**Figure 3:**
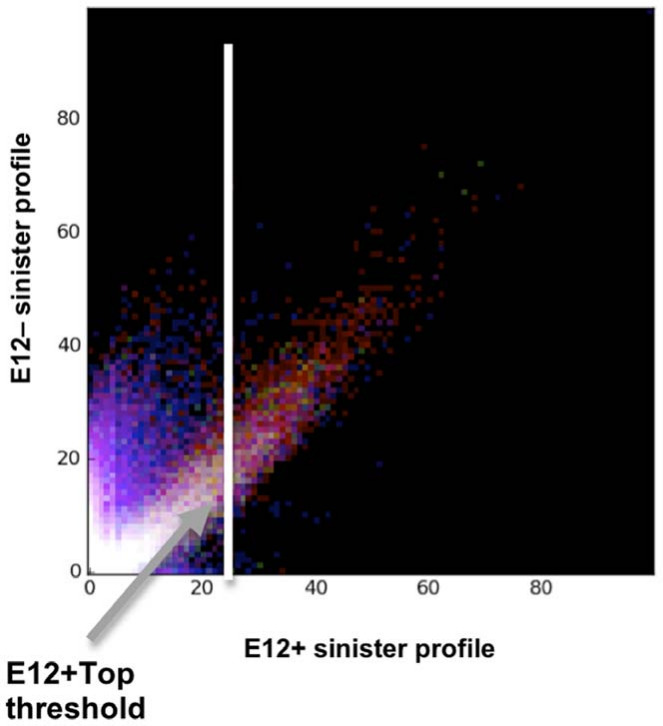
Classification of Reads. Counts from the merged profiles (E12− and E12+) were paired in a coordinate and strand-sensitive manner at every location in the genome. The 3-way classification scheme was used to partition the pairs into subsets containing paired values from annotated (blue), opposite (green) and intergenic regions (red). All pairs containing one or two zero counts were discarded. Because the dynamic range of counts is so large, each value was incremented by 1 and log(10) of both values was calculated. Pairs from each of the three subsets were separately binned using a 100×100 grid. The grids were normalized by dividing the counts in every cell by the largest cell count in all three grids. Each grid was assigned a color (red, green or blue) with cell values establishing color intensity. The grids were then layered to produce a full-color density plot. The extremely high cell values in the vicinity of the origin were set to zero prior to normalization to boost the visibility of cells with small count values. This produces the black square in the lower left corner. The white line represents the threshold used to select the E12+Top.

Although the above analysis effectively captures the bulk properties of the data, it is difficult to discern how the different sub-populations responded to exonuclease treatment. So, to determine an appropriate threshold for subsequent analyses, we compared the 5′-ends from each of the two treatments in a scatter plot after normalizing total counts by scaling and binning (Figure 3) and used the information concerning the expected behavior of the 5′-ends in the presence or absence of exonuclease and the location of the 5′-end (annotated, opposite, intergenic) to guide selection of a threshold. This approach reveals additional structure. The scatter plot shows that it is difficult to determine if those captured 5′-ends with very low counts in both treatments (near the origin) display sensitivity to exonuclease treatment. The brightness in this region indicates that many captured 5′-ends share this property. However, as one examines captured 5′-ends in the E12− sample that have increasing counts (in effect, by moving a horizontal line up the plot away from the x-axis, and focusing only on points above the line), the distribution appears to resolve into exonuclease sensitive and resistant cohorts. First, closer to the y-axis are 5′- ends with high count numbers in E12− but much lower count numbers in E12+. These are 5′-ends that are sensitive to exonuclease treatment. Second, to the right are ends that fall along a distinct diagonal. These are 5′-ends that resist exonuclease treatment and are present in roughly equal number of counts in both treatments. The color (red) of the 5′-ends within the diagonal indicates that this region is enriched for 5′-ends in intergenic regions, as expected.

Because the diagonal contains 5′-ends that are most likely associated with transcription initiation events, we selected this cohort for additional analyses. The final cutoff value (determined impressionistically) was set at bin 22 on the y-axis. This also corresponds to a cutoff at bin 22 on the x-axis since the plot is normalized. The placement of a vertical line at this location effectively isolates ends in the diagonal from nearly all others on the plot (those with low numbers of counts and those 5′-ends which were most impacted by treatment with exonuclease), allowing us to focus on a relatively small data set, designated E12+Top. The cutoff corresponds to a cutoff of 118 counts in the E12+Top data set, yielding approximately 2500 captured 5′-ends. We emphasize that this cutoff is intended to provide high-quality targets rather to define a level for true TSSs, and is likely to exclude legitimate TSSs (see below).

With the threshold set, the number of positions for each of the classifications for ends with counts greater than the selected threshold was determined. The majority of 5′-ends (1669) in the E12+Top fell in intergenic regions, 616 5′-ends fell within gene annotations (annotated), and 225 fell with gene annotations on the “wrong” strand (opposite). Of the intergenic peaks, 86 are located in convergent regions, 751 are located in divergent regions and 832 are located in co-stranded regions.

### Comparison with global RNA-Seq data

The 5′-end capture experiments were performed using RNA isolated from bacteria grown under the same conditions as those used in our stranded global RNA-Seq experiment [17].

As expected we detected transcriptional start sites for genes previously found to be expressed under iron-limited conditions, such as those involved in iron uptake, iron binding, and iron transport [21]. We also detected transcriptional start sites for genes that encode known or predicted virulence-related genes [1], [24], including those involved in type III secretion (T3S) and coronatine production.

To determine if the 5′-ends found in the current experiment coincide with the abrupt transcription level transitions observed using global RNA-Seq, we first examined the number of reads in the stranded RNA-Seq sinister profiles surrounding each captured 5′-end (+/− 10 bps). Profile counts on either side of all 2482 captured ends in E12+Top were compared and a ratio was computed. The histogram of the ratios shown in Figure S2 shows that, on average, the sampled regions downstream of 5′-ends have 3.61 times more reads than the corresponding regions upstream, consistent with transcript initiation at many of these sites. Using computer simulation, we found that the probability of 2482 randomly chosen genomic positions having a mean ratio of 3.61 or greater was effectively zero (p-value<2e-6 (1/500000)). We then used a sliding window analysis to look more closely at the relationship between the transition from few to many counts in the RNA-Seq data and the position of the 5′-ends (Figure S2). The results show that the RNA-Seq transition is coincident with the 5′-ends from the E12+Top dataset. In some cases the ratio was <0.5, suggesting higher levels of transcription upstream of the position. A majority of these represented 5′-ends located within annotated genes. These cases may represent active promoters embedded within transcriptionally active regions.

We previously reported 11 new DC3000 genes that corresponded to locations in the DC3000 genome where we observed transcriptional activity inconsistent with genome annotation [17]. The 5′-end capture data in these areas is consistent with the new gene calls for PSPTO_5635, PSPTO_5637, PSPTO_5638 and PSPTO_5644, supporting the view that high-throughput 5′-end capture reliably identifies areas in the genome where transcription is not consistent with the annotation.

It has been reported that 46% of the RNAs with published start sites initiate with “A” in E. coli [12] and it is generally accepted that sigma70 promoters tend to initiate transcription with A or G [25]. To determine if there is a bias for a particular nucleotide in the captured RNAs, a count of the base that appears at the first position of each of the 2510 captured ends in the E12+Top dataset was calculated. 981 transcripts (39%) initiate with an “A”, 735 (29%) with a “G”, 345 (14%) with a “T” and 449 with a “C” (18%). The predominance of A and G termini (68%) is consistent with reports for *E. coli* and *Synechocystis* transcripts [10], [16].

### Length of 5′–UTRs

Several recent analyses evaluating transcriptional start sites on a global scale report the distance between transcriptional start sites and annotated translation start codons [11], [13], [15], [26]. To determine the average length of the 5′-UTR for DC3000, we evaluated those 5′-ends classified as intergenic and located within 1000 bases of the downstream annotated CDS. The mean distance of the 5′-UTR was 77 nucleotides (Figure S3) and is slightly longer than the 5′-UTR lengths reported for other bacteria using a variety of methods [11], [13], [15], [26].

Next we evaluated 5′-ends that fell within the first 100 bases of a gene (97 cases) to determine if their paradoxical position was due to start codon mis-annotation. 56 of the 97 ends had promoter-like elements (see below) associated with them, consistent with transcription initiation at these locations. One example is PSPTO_3157. The location of the mapped transcriptional start site for PSPTO_3157, along with an adjacent Fur-binding site suggest, that the annotated CDS is too large [17], [27]. The other cases may also represent candidates for re-annotation.

### Agreement with previously reported transcriptional start sites

Previously, we identified over 60 transcriptional starts sites using conventional 5′RACE [17]. Forty-eight out of 66 correspond to the 5′-ends captured using the high-throughput method. If we extend the analysis to include those captured 5′-ends with counts below the E12+Top threshold, nearly all (65/66) matched in the larger E12+ dataset. To further extend the comparison between methods, 5′RACE was used to determine the start sites for 27 genes thought to be regulated by RpoF. Sixteen 5′-ends matched perfectly with transcriptional start sites in E12+Top (Tables S3 and S4) and all 27 matched in the larger E12+ dataset, which includes captured 5′-ends with counts below the E12+Top threshold.

Overall, these results confirm the correspondence between 5′-end capture and 5′RACE, and also demonstrate that some legitimate TSSs with relatively low counts were omitted from the E12+Top data set.

### Association of 5′-ends with promoter motifs

**Figure 4:**
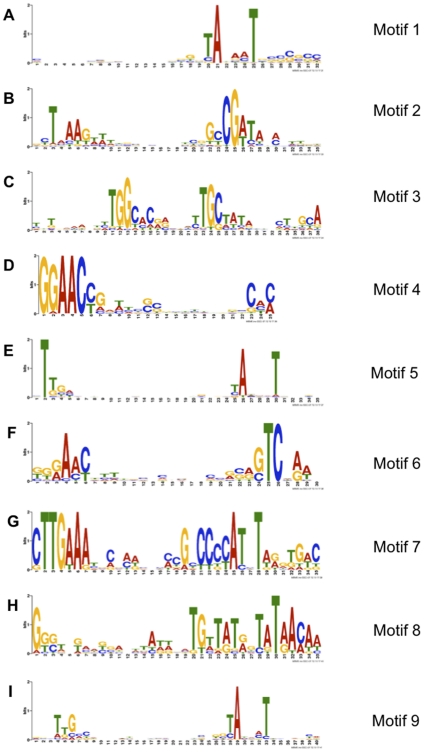
Sequence motifs identified by MEME. Sequence logos of nine motifs generated by MEME [28]. MEME motifs are represented by position-specific probability matrices that specify the probability of each possible letter appearing at each possible position in an occurrence of the motif. The sequence LOGOS contain stacks of letters at each position in the motif. The height of the individual letters in a stack is the probability of the letter at that position multiplied by the total information content of the stack. The height of a letter indicates its relative frequency at the given position (x-axis) in the motif.

Genuine transcription start sites should often occur in close proximity to upstream promoters, while ends arising from other events would not be expected to have this relationship. To identify putative promoter motifs, we sampled 40 nucleotides upstream of the 5′-ends collected in E12+Top, resulting in 1827 sequences (overlapping samples were merged). The samples were used as input to the *de novo* motif discovery tool Multiple EM for Motif Elicitation (MEME) [28] to find over-represented motifs. Nine motifs (E-value of <1) with structures suggestive of promoters were detected in 1214 of the input sequences (Figure 4). In many cases these resembled promoters already reported in the literature for DC3000 or other bacteria. Approximately one-third (613) had no identifiable promoter element using MEME, but some promoters may not be sufficiently conserved or abundant to be detected in this analysis. In contrast, MEME analysis of 1827 randomly selected sequences of similar size (length) from the DC3000 chromosome yielded no recognizable promoter elements (data not shown).

#### Motif 2

(Figure 4B; 76 sequences) is very similar to the consensus promoter motif recognized by RpoF (TAAAAGN_15_ GCCGATA) [29]. RpoF, also known as FliA or sigma 28, is responsible for the expression of flagellin in *P. aeruginosa* [29], [30]. As expected, this motif was associated with *fliC* (PSPTO_1949) in *P. syringae*, as well as a number of genes predicted to encode for proteins involved in chemotaxis (Dataset S12). This motif was also found upstream of two conserved proteins of unknown function (PSPTO_5635 and PSPTO_5638), which have recently been added to the DC3000 genome annotation. The motif was consistent with 14 of the transcriptional start sites determined by 5′RACE (reported above) for putative RpoF regulated genes. Some sequences contributing to motif 2 are likely promoters recognized by PvdS (see below).

#### Motif 3

(Figure 4C; 39 sequences) resembled the consensus promoter motif recognized by RpoN or sigma 54 (−24(GG)/ −12(GC) [30], [31]. RpoN plays important roles in motility, formation of pili, in mucoidy, and cell-to-cell signaling (reviewed in [30]). In *P. syringae* RpoN has been reported to regulate HprL, the alternative sigma factor responsible for expression of the many of the genes of the Type III secretion system [32]. This motif was present upstream of the transcriptional start site for *hrpL* as well as *hcp-2*, a protein secreted by the recently described Type VI secretion system, and several ncRNAs that are reported to be regulated by RpoN [17](Dataset S12).

#### Motif 6

(Figure 4F; 47 sequences) is very similar to the promoter sequence recognized by RpoE, AlgU or sigma 22 (GAACTT-N_16–17_-TCcaA) [33]. Interestingly, this motif is found upstream of a recently described ncRNA, PSPTO_5670, in DC3000 [17]. RpoE/AlgU is involved in the conversion of *P. aeruginosa* from non-mucoidy to mucoidy and resistance to oxidative stress [30]. In *P. aeruginosa*, 35 genes are predicted to be directly regulated by AlgU [33]. Most of the genes in downstream of motif 6 encode hypothetical proteins. However, we found that motif 6 was present upstream of PSPTO_4224 (RpoE/AlgU) suggesting autoregulation, as shown in *P. aeruginosa* [34], [35].

#### Motif 7

(Figure 4G; 8 sequences) resembles the RpoH motifs (CNCTTGAAA-N_13–14_-CCCCATNT) [36] and GGCTTGA-N_12–20_-CCCCAT-3′ [37]. RpoH is required for the positive regulation of heat-shock genes and heat-shock proteins [38]. All eight captured 5′-ends associated with this motif precede proteins involved in the heat shock response (Dataset S12).

#### Motif 8

(Figure 4H; 13 sequences) contains an inverted repeat and in two cases appears in regions where there are two overlapping divergent promoters (PSPTO_5559/PSPTO_5560) and PSPTO_5526/PSPTO_5527). Interestingly, the most conserved portion of this motif contains a 7-1-7 inverted repeat (TGTTATA/G_TATAACA), which is characteristic of the sequences recognized by members of the Fur (ferric uptake regulator) superfamily (Zur, Per, Fur) [39] and resembles the Zur binding motif for γ-proteobacteria (GAAATGTTATANTATAACATTTC) reported in Panina, EM [40]. Since several genes downstream of the motif appear to be involved in zinc homeostasis and are regulated by Zur in other organisms [41], [42], we hypothesize that this motif is recognized and bound by Zur. Further studies will be required to determine if Zur protein binds at these locations.

#### Motifs 1, 5 and 9

(Figure 4A, E, and I; 1002 sequences) resemble the RpoD promoter element and are extremely similar to the RpoD motif for *P. syringae* reported by Bronstein *et al.* [21]. Sigma 70 promoters would be expected to dominate the data set because RpoD is the sigma factor associated with the expression of “housekeeping” genes. However, the motif we report here has relatively low information content and should be interpreted cautiously. The canonical model of the RpoD promoter element is defined as a −35 hexamer (TTGACA) and a −10 hexamer (TATAAT), separated by 15–21 base pair spacer [43]. The motifs generated by MEME contained a pronounced −10 site, but no obvious −35 hexamer, except for a prominent “TGG” in two of the three motifs. In *E. coli*, the first three nucleotides of the −35 hexamer (TTG) are more conserved than the other nucleotides [44]–[47]. Included in this set is a transcriptional start site embedded approximately 10 bases downstream of the start codon for PSPTO_4724 (*hopD*), which is HrpL-regulated [18]. Because upstream sampling for MEME was limited to 40 bp from the captured 5′ ends, the HrpL-dependent promoter further upstream of this site (−60 to −91) was not included in the input data set.

### Cross validation with HrpL-dependent promoters

#### Motif 4

(Figure 4D; 42 sequences) highly resembles the HrpL-dependent promoter GGAAC-N(x)-CCAC in Ferreira *et al.* that regulates 51 operons in DC3000 [18]. Twenty-six of the 51 HrpL-dependent promoters are at locations consistent with transcription initiation at captured 5′-ends in the E12+Top data set and all 26 are included within the aligned motif 4. However, motif 4 includes 16 other sequences that are similar to those in motif 6 (resembling RpoE/AlgU). Although the 16 sequences included in motif 4 which we do not believe to be HrpL-dependent, do not posses the strongly conserved −10 box (CCAC) characteristic of HrpL-dependent promoters, they have similar −35 boxes (GAAC). Therefore, some mingling between motifs 4 and motif 6 presumably occurs. Also, the HrpL-dependent promoters identified in Ferreria *et al.* have variable spacing between the −35 and −10 boxes (GGAAC-N_16–17_-CCAC), and because MEME does not produce gapped alignments, the alignment appears to be restricted to the -35 box and motif 4 does not reveal the strongly conserved −10 box (CCAC). Closer examination of motif 6 reveals that many of the 16 non-HrpL-related sequences contain GTC in the −10 box rather the CCAC characteristic of the HrpL promoter. This shared feature may account for the co-clustering.

**Figure 5:**
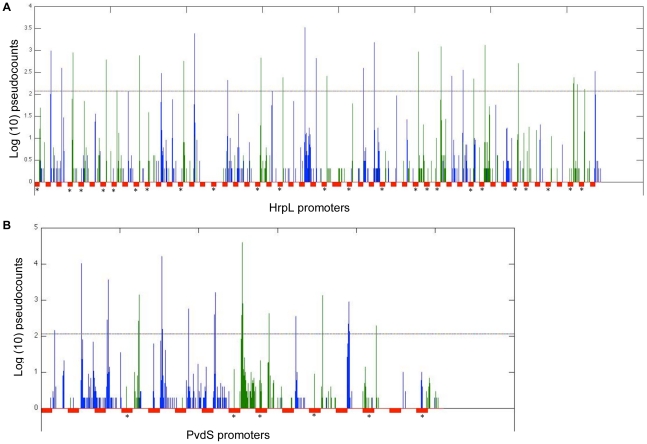
5′-ends associated with the HrpL and PvdS promoter motifs. Composites of all of the annotated HrpL (**A**) and those PvdS (**B**) promoters exhibiting PvdS-dependent expression were constructed. Each promoter is represented below the X-axis by a red box and is oriented 5′ to 3′, left to right. Above the X-axis are spikes representing log(10) pseudocounts for captured 5′ ends at the corresponding genomic coordinates. The counts are derived from Supplemental Dataset S6. Green spikes are counts on the negative strand and blue spikes are on the positive strand. The promoters and their downstream regions are presented one after another with no intervening space. Promoters located on the negative strand are denoted with asterisks (*). The horizontal line just above Y = 2 is the cutoff (118 counts).

To determine whether there are some 5′-ends associated with HrpL-dependent promoters below our cut-off, we performed a census of captured 5′ ends within annotated HrpL-driven promoters and in their downstream (40 bp) sequences. The results show that in fact all except two (49/51) HrpL-dependent promoters have TSS associated with them (Figure 5A). Surprisingly, the detailed inspection also revealed that these promoters were almost always associated with a group (or cluster) of closely spaced TSSs rather than a single isolated 5′-end. Typically only one position in each cluster exceeds the cutoff.

### Cross validation with PvdS-dependent promoters

Although the RNA for this experiment was isolated from bacteria grown under iron-limited conditions, MEME did not identify a distinct motif that resembles the PvdS promoter element [19]. In Swingle 2008, 15 operons exhibited PvdS-dependent expression and were associated with a “PvdS box”, i.e., a sigma factor-DNA binding site. Thirteen of the 15 PvdS-dependent promoters lie sufficiently close to the 5′-ends in the E12+Top data set and were included in the sequences for the MEME input. Nine of the 13 promoter sequences are associated by MEME with the motif 2 (which resembles RpoF). The −35 boxes of PvdS (TAAATA) and RpoF (apparently associated with motif 2, TAAAAG) are similar, which is perhaps why the PvdS-dependent promoters have been clustered in motif2. The PvdS −10 box, however, contains a highly conserved CGTT motif, which is not found in most of the rest of the sequences that are clustered in motif 2. The remaining four PvdS-regulated promoters with accompanying transcriptional start site did not appear in any of the other motif groups generated by the MEME analysis.

When we evaluate all positions below our threshold, all (15/15) PvdS-dependent promoters had captured 5′-ends downstream of the promoter motif (Figure 5B). There were a few coordinates within several promoters themselves that had counts. However, the magnitude of these counts was 1 (equal to 0.3 on the Y-axis). As seen with the HrpL-dependent promoters, clusters of 5′-ends were also associated with PvdS-dependent promoters.

### Captured 5′-ends and operon predictions

With our global survey of putative TSSs we can compare those results with genome-scale computational predictions of operons in DC3000. If the 5′-ends detected reflect true TSSs, then we would expect those start sites generally to be located upstream of operons. Efforts to predict operons in bacterial genomes consider all possible operon junctions (i.e., all pairs of adjacent annotated genes lying on the same strand) and assess whether that gene pair is more likely to be part of a single operon (operon pair) or part of separate operons (boundary pair). We examined the 2510 captured 5′-ends in the E12+Top data set using a set of predictions for DC3000 from the microbesonline.org resources provided by the Virtual Institute for Microbial Stress and Survival (VIMSS;www.microbesonline.org/operons/gnc223283.html) [48].

For the main chromosome of the DC3000 genome, VIMSS predicts 1019999 of 12,794,252 positions (7.9%) to lie in the upstream region of the predicted operons. A captured end was considered to be “associated with an operon if it fell within a window extending 250 bases upstream and 50 bases downstream of the 5′-end of the first gene of a predicted operon. We found that 1456 of the 2510 (E12+Top) (58.0%) TSSs lie upstream of the predicted operons. Using both a one-tailed binomial test and a normal approximation to the binomial test approaches for large samples sizes, we determined that the probability of observing this result by chance was essentially zero (p-values too small to compute due to numerical underflow).

To determine the occurrence of multiple TSSs in DC3000 the number of TSSs per operon was computed. According to the VIMSS operon predictions, DC3000 is predicted to have 3417 operons, yielding an average of 1.6 genes per operon. We found that 942 operons had at least one 5′-end before the first gene of the operon. 1456 5′-ends (from the E12+Top dataset) were located in front of the first gene of the predicted operons, yielding an average of 1.5 5′-ends per operon. Most operons have just one 5′-end (623) associated with them. Of the operons that have multiple 5′-ends preceding the first gene (319), some possess multiple MEME-predicted motifs. For example, PSPTO_5162 (*mdoG*) has a predicted RpoD promoter (motif 1) and a putative RpoE-like promoter. Other examples include, PSPTO_1377 (*avrE1*) which has a predicted HrpL promoter as well as an RpoD-like (motif 1) promoter and PSPTO_5251 (*hemB*) which has two RpoD-like promoters (motif 1 and 5) 47 bases apart from one another.

### Presence of overlapping promoters

We examined promoters whose proximity and orientation may lead to interference, focusing on MEME-predicted motifs that are located within 100 bases of one another regardless of strand (direction). We found 103 cases that satisfy these criteria and in 17 of these the promoter motifs actually overlap (by at least 1 bp). Since our analysis focuses only on TSSs for which we can identify an accompanying promoter motif, it provides a low-end estimate for promoters positioned in these ways.

### Evidence for antisense activity

**Figure 6:**
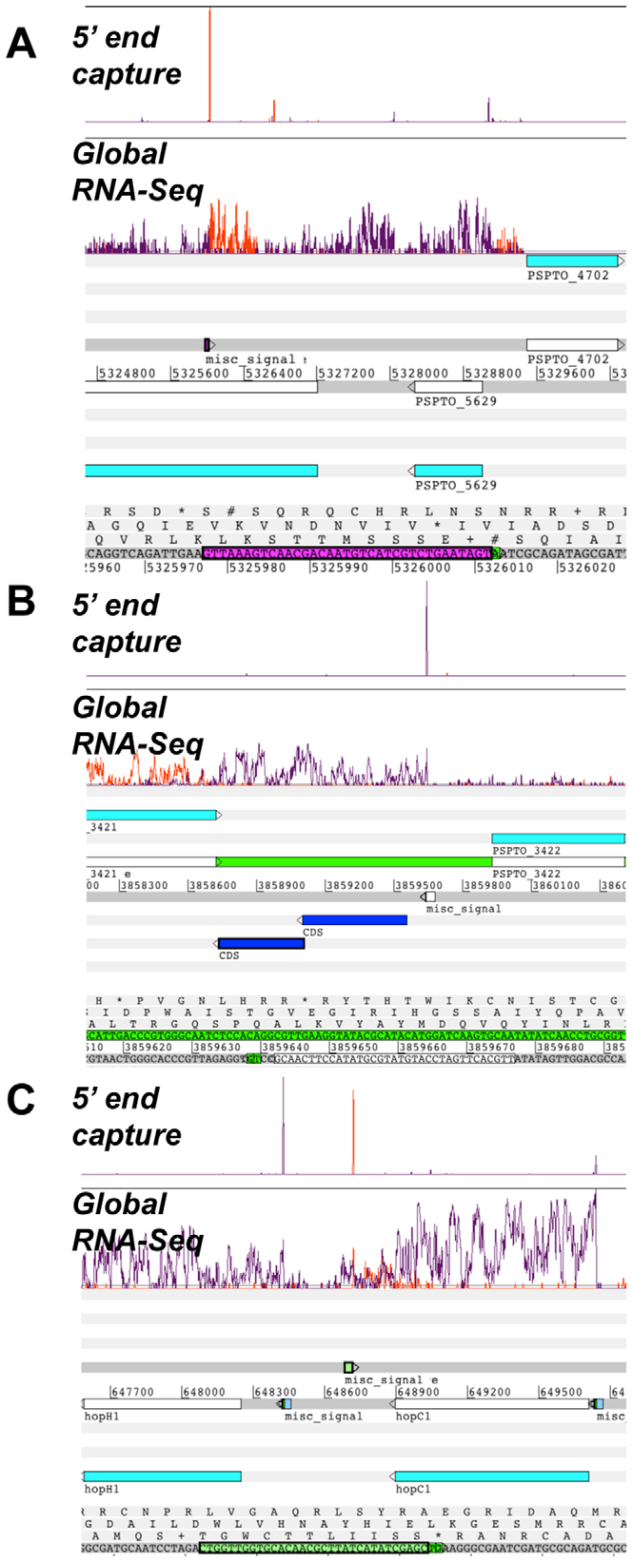
Profiles of transcriptional activity displayed using Artemis. (A) represents an examples of putative antisense activity. (B) depicts an example of a putative new CDS and (C) is an example of a putative non-coding small RNA. The transcriptional profile (orange and purple) is shown above the annotated genome. An orange trace indicates transcription from left to right (on the positive strand) and a purple trace represents transcription from right to left (on the negative strand). In panel (A), the magenta colored misc_signal and highlighted sequence represents a putative promoter sequence. The nucleotides highlighted green are confirmed transcriptional start sites. IN panel (B) the blue CDSs represent EasyGene calls. In panel (C), the misc_signals (blue and green) correspond to predicted promoter sequences identified through the MEME analysis.

225 captured 5′-ends from E12+Top were located within a gene, but appear to arise from transcription on the non-coding strand (opposite strand). We compared these “antisense ends” with the global RNA-Seq data and found 44 5′-ends, corresponding to 30 genes that displayed the same antisense activity (classified as red or yellow in Filiatrault *et al.* [17]) (Table 2). 27 of these had recognizable promoter motifs associated with them. Genes exhibiting antisense activity include PSPTO_0044 (*hopK1*), PSPTO_0852 (*hopAJ1*), PSPTO_2862, and PSPTO_4302 (Figure 6A). Interestingly, Swingle *et al.* previously detected a predicted a PvdS binding site antisense to PSPTO_2862 [19]. Many other (100) antisense ends were associated with genes that were expressed in a manner consistent with annotation (green in Filiatrault *et al.* [17]). Examples include antisense activity associated with PSPTO_3566 (*csrA-3*), and PSPTO_2830 (*syfB*). A number of these cases had recognizable promoter motifs associated with them. These results provide additional evidence for antisense activity in DC3000.

**Table 2:** Evidence for antisense activity.

	PSPTO# b	gene	MEME motif
***5′-ends in genes colored red or yellow*** a	PSPTO_5522		motif1
	PSPTO_5306		
	PSPTO_4614		motif5
	PSPTO_4541		motif 5
	PSPTO_4509 (2)	*omlA*	motif1
	PSPTO_4456		motif5
	PSPTO_4385 (2)		
	PSPTO_4383		
	PSPTO_4322		motif1
	PSPTO_4302		
	PSPTO_4150		
	PSPTO_3673 (2)		motif 6
	PSPTO_3578		motif5
	PSPTO_3483		motif5
	PSPTO_2862 (6)		motif5, motif 2
	PSPTO_2356		motif1
	PSPTO_2227 (2)		motif2
	PSPTO_2194	*gltA*	
	PSPTO_1938		
	PSPTO_1935	*flgD*	motif9
	PSPTO_1595		motif1
	PSPTO_1591		motif1
	PSPTO_1321		motif3
	PSPTO_0852	*hopAJ1*	
	PSPTO_0848		
	PSPTO_0803 (2)		motif1
	PSPTO_0607		motif5
	PSPTO_0416		motif5
	PSPTO_0336		motif9
	PSPTO_0044 (4)	*hopK1*	
***5′-ends in selected genes colored green***	PSPTO_5391		
	PSPTO_5345		
	PSPTO_5331		
	PSPTO_5251	*hemB*	motif 1
	PSPTO_5133		motif 3
	PSPTO_4932		motif 5
	PSPTO_4699		motif 2
	PSPTO_4286		motif 9
	PSPTO_3566	*csrA-3*	motif 5
	PSPTO_3529		
	PSPTO_3370	*nouG*	motif 5
	PSPTO_3105		motif 6
	PSPTO_2830	*syfB*	
	PSPTO_1660		motif 9
	PSPTO_1074		motif 1
	PSPTO_0803		motif 1
	PSPTO_0537	*rpoD*	motif 5
	PSPTO_0087		motif 1

a red, yellow, and green refers to classification in Filiatrault *et al.*, 2010.

b If more than one 5′-end was associated with the gene, parentheses with the number of 5′ends are shown.

Since “antisense” ends might in some cases be associated with genes on the sense strand further downstream, the distance from the putative antisense TSSs to the first annotated ORF (co-stranded with the 5′-end) was calculated. We found only 38 of the 225 “antisense” ends to be within 250 bases of a corresponding gene on the same strand of the 5′-end. These potentially represent TSSs for these genes.

### Identification of new CDSs

Since the DC3000 genome was originally annotated using GLIMMER [49], we applied a different gene finder program (EasyGene version 1.2; [50]) to determine whether some of the captured 5′-ends located in large intergenic regions might be associated with unannotated genes. We identified 14 potential CDSs with associated TSSs (Table 3), nine of which were associated with putative promoters identified by MEME. For example, two new CDSs are located between PSPTO_3421 and PSPTO_3422, which is in a larger feature annotated as Phage03 [1]. These CDSs are predicted to encode for hypothetical proteins (Table 3 and Figure 6B). Another putative CDS, located in an intergenic region between PSPTO_1132 and PSPTO_1133, encodes for a conserved hypothetical protein, which is annotated in both *P. syringae* 1448A and *P. syringae* B728a. The three genes in *P. syringae* 1448A and *P. syringae* B728a appear to comprise an operon (www.pseudomonas.com). However, the position of the 5′-end in DC3000 suggests that the last gene of the operon can be transcribed independently. Interestingly, the upstream region of another CDS located between PSPTO_3948 and PSPTO_3049 was recently shown to contain a binding site for Fur (ferric uptake regulator) [27], suggesting its expression is dependent on iron concentrations.

**Table 3:** Identification of new CDSs.

Ig region^a^	EasyGene coordinates (nt)	Description of EasyGene call^b^	MEME motif
PSPTO_0571/PSPTO_0572	628937..629374	hypothetical	motif 5
PSPTO_0666/PSPTO_0667	720426..720668	hypothetical	
PSPTO_0668/PSPTO_0669	722904..723323	putative lipoprotein	motif 6
PSPTO_1454/PSPTO_1455	1596770..1597255	hypothetical	motif 1
PSPTO_4626/PSPTO_4627	5222736..5223122c	hypothetical	motif 9
PSPTO_3984	4490601..4491209	cold shock DNA binding protein	motif 5
PSPTO_3948/PSPTO_3949	4456836..4457390	hypothetical	motif 1; motif 6
PSPTO_3421/PSPTO_3422	3859101..3859553c	hypothetical	motif 5
PSPTO_3421/PSPTO_3422	3858736..3859104c	hypothetical	
PSPTO_3208/PSPTO_3210	3603655..3604008c		
PSPTO_3208/PSPTO_3210	3603391..3603639c		
PSPTO_2390/PSPTO_2391	2640416..2640622		
PSPTO_0287/PSPTO_0288	315392..315895c	hypothetical	motif 5
PSPTO_0023/PSPTO_0024	30005..30553c	COG4127	motif 5

a The CDSs flanking the 5′-end.

b Results of BLAST analysis of predicted protein from EasyGene call.

### Candidate small non-coding RNAs

Some 5′-ends were classified as “intergenic” but did not appear to be plausibly associated with an annotated gene because the closest gene was relatively distant, the end was oriented inappropriately, or another 5′-end was more proximal to the gene. We entertained the possibility that these “orphaned” 5′-ends might represent transcriptional start sites for small non-coding RNAs (ncRNAs). Captured 5′-ends for a number of recently identified ncRNAS, such as *rsmZ*, *rsmY*, *rsmX1-X5*, *psr1*, *psr2*, and *spot 42* were consistent with reported transcriptional start sites [17], [51]. Other orphaned-ends were positioned just upstream of transcriptionally active areas that were detected by global RNA-seq. The newly identified candidate ncRNAs and their putative promoters are listed in Table 4. Several appear to have Rho-independent terminators, providing further evidence that these transcripts may represent ncRNAs. Interestingly, the region between PSPTO_0588 (*hopH1*) and PSPTO_0589 (*hopC1*) appears to encode a ncRNA (Table 4 and Figure 6C).

**Table 4:** Putative non-coding RNAs.

IG region^a^	MEME motif
PSPTO_4734/PSPTO_4735	
PSPTO_4696/PSPTO_4697	
PSPTO_4675/PSPTO_4676	motif 1
PSPTO_4630/PSPTO_4631	motif 1
PSPTO_4628/PSPTO_4629	motif 3
PSPTO_4545/PSPTO_4546	
PSPTO_4426/PSPTO_4427	motif 1
PSPTO_4310/PSPTO_5644	motif 5
PSPTO_4299/PSPTO_4300	motif 9
PSPTO_3986/PSPTO_3987	motif 3
PSPTO_3930/PSPTO_3931	motif 9
PSPTO_3805/PSPTO_3806	
PSPTO_3653/PSPTO_3654	
PSPTO_3565/PSPTO_3666	
PSPTO_3480/PSPTO_3481	motif 4
PSPTO_3477/PSPTO_3478	motif 5
PSPTO_3332/PSPTO_3333	
PSPTO_3156/PSPTO_3157*	
PSPTO_3145/PSPTO_3146	
PSPTO_2856/PSPTO_2857	motif 5
PSPTO_2855/PSPTO_2856	
PSPTO_2851/PSPTO_2852	
PSPTO_2528/PSPTO_2529	motif 5
PSPTO_2440/PSPTO_2441	motif 2
PSPTO_1825/PSPTO_1826	motif 1
PSPTO_1820/PSPTO_1821	
PSPTO_1721/PSPTO_1722	motif 1
PSPTO_1662/PSPTO_1663	motif 1
PSPTO_1592/PSPTO_1593	
PSPTO_1136/PSPTO_1137	motif 1
PSPTO_1083/PSPTO_1084	
PSPTO_1082/PSPTO_1083	motif 1
PSPTO_0588/PSPTO_0589	motif 1
PSPTO_0529/PSPTO_0530	
PSPTO_0526/PSPTO_0527	motif 1

a The CDSs flanking the 5′-end.

*antisense to PSPTO_5657 (ncRNA *prrF2*).

## Discussion

Bacterial transcriptional start sites have typically been identified using primer extension, S1 nuclease mapping, and 5′RACE. These methods are labor intensive and have limited sensitivity. However, their primary drawback is their inability to recognize internal transcriptional start sites or to differentiate between processed 5′-ends and primary transcripts. Recently, RNA-Seq has been adapted to address these issues. One powerful approach (dRNA-Seq) employs the enzyme terminator exonuclease to differentiate between primary and secondary processed transcripts by selectively degrading processed RNAs. For example, Sharma [15] was able to infer the existence of many 5′-ends from the relative increase in reads at particular genomic locations after treatment by exonuclease.

The method we report here also exploits this enzyme, but our method increases sensitivity by specifically targeting 5′-ends, leaving the remainder of the transcriptome unsequenced. This refinement enables the detection of 5′-ends even when they are embedded in transcriptionally active areas. Our conservative analysis focused on approximately 2500 captured 5′-ends with the highest read counts and only considered ends that resisted degradation by the terminator exonuclease. Since conversion of the 5′-triphosphate to a 5′-monophosphate is the first step in mRNA degradation [52], [53], exonuclease treatment may eliminate some otherwise intact mRNAs that exist in the 5′-monophosphate form within the cell, but we did not investigate this further.

We combined information from captured 5′-ends with an independently generated global transcript map of DC3000 determined under identical conditions, allowing us to perform a comprehensive assessment of newly identified 5′-ends. A large majority of previously identified transcriptional start sites (i.e., using 5′RACE) matched 5′-ends captured using our method. This consistency boosts our confidence that our method captures *bona fide* 5′-ends, including those that appear to be associated with antisense transcripts, ncRNAs, and new CDSs. Overall, we report more than 1200 putative transcriptional start sites for *P. syringae* DC3000. It is important to note that our analysis excludes otherwise legitimate TSSs that did not have sufficient numbers of sequence reads to meet our cutoff. Our survey should therefore be viewed as a low estimate of promoter abundance.

Using a tightly localized search upstream of the identified 5′-ends we were surprised to discovered nine motifs representing putative promoter or other regulatory elements. Other RNA-Seq studies investigating transcriptional start sites have reported far fewer putative promoters. Our decision to limit motif discovery to ends with relatively high read counts may have been a contributing factor, and we note that the selection of MEME parameters can profoundly effect motif discovery. For example, a MEME analysis biased toward palindromic (inverted repeat) sequences recovered a motif recognized by Fur (ferric uptake regulator) an important regulator of iron homeostasis [27] and [21]); 18 out of 20 of these locations have ChIP-Seq peaks associated with them [27]. This pattern was not detected in the MEME analysis that recovered the other motifs reported here.

In many cases the motifs clearly resemble consensus sequences for regulatory elements reported for *P. syringae*, and other Pseudomonads [18], [19], [30]. Motif 2, and motif 4 appear to be composites that can be attributed to well described sigma factors. For example, sequences upstream of genes regulated by the sigma factor PvdS were clustered with a motif that more closely resembles a promoter consensus sequence described for RpoF. Due to high sequence similarity between the sequences recognized by these sigma factors, MEME may be unable to differentiate the two motifs given the parameters we used. We emphasize that the promoters we report here are putative and should be regarded as working hypotheses. Further investigations will be required to demonstrate that they function as authentic promoters. Nonetheless, these tentative assignments provide an initial step towards a comprehensive regulatory analysis of *P. syringae* and will be particularly interesting when integrated with additional transcript profiles and data obtained for sigma factor binding by chromatin immunoprecipitation (ChIP-Seq). The ideal approach has been described for *Geobacter* [11].

Because genes in bacterial genomes are arranged in monocistronic or polycistronic operons, the analysis of transcript structure is fundamental to the study of gene regulation. The *P. syringae* DC3000 genome has approximately 5600 annotated genes [1] in more than 3400 predicted transcriptional units [48]. Although it is difficult to predict the number of “capturable” 5′-ends under particular growth conditions, we believe the range may extend from several hundred to several thousand, depending on assumptions about the number of active genes, the presence of alternative promoters regulating a single transcription unit, and the frequency of anomalous transcription initiation events within genes or in antisense orientations. An exhaustive inventory would require multiple growth conditions. Regardless, our study indicates that transcriptional start sites are generally not located between genes predicted to be part of an operon and is in agreement with results reported for *B. anthracis* [54]. Although analyses like these will help guide future experiments, the comprehensive investigation of operon structure will require longer read lengths that span entire transcriptional units.

A large fraction (42%) of TSSs are not located immediately upstream of operons. There are several possible explanations for this result. One contributing factor is that operon predictions are likely to contain inaccuracies that obscure the spatial relationship between TSSs and operons. Operon prediction is an evolving field, and predictions made using alternative methods for *P. syringae* DC3000 show only 86% overlap. These predictions have not been validated as few operons in DC3000 have been experimentally verified. In addition, since operon prediction depends at least in part on pre-existing annotation, it includes other sources of error such as miscalled genes, incorrect placement of translation start sites, and missed ORFs.

A second factor is that some 5′-ends may arise as a consequence of antisense transcription. Recently substantial numbers of antisense RNAs have been reported in bacteria (reviewed in [55] and [12], [15], [16], [56]). We report 225 ends whose positions are consistent with this property. It is possible that some of these are in fact upstream of genes encoded on the same strand. 38 of the 225 TSSs are within 250 bases of a corresponding gene on the same strand of the 5-end and can be considered as canonically positioned with respect to those genes. However, this leaves a substantial number of antisense TSSs (187) without an apparent associated gene, and these will be excellent candidates for future investigation into antisense transcription. Since we see considerable agreement using global RNA-Seq [17], 5′-end capture (this study) and small RNA-Seq (unpublished), we are confident that these represent real transcriptional events. However, it remains to be determined whether antisense transcripts are involved in transcriptional interference, modulate translation, or influence transcription termination [55].

It is also possible that some TSSs are in fact adjacent to unannotated protein-coding genes or small non-coding RNAs. Our definition of operon association requires that a 5′-end must fall within 250 nucleotides upstream or 50 nucleotides downstream of the start codon of the first gene of an operon. TSSs (565) that are too far upstream (>250 bases) to be associated with an operon appear to fall in regions that are empty of annotated objects. However, improvements in annotation may populate much of this real estate, revealing that these TSSs are positioned in the ordinary way relative to their target genes.

Finally, the remaining TSSs (489; 19.4%) fall within operons on the sense strand but more than 50 nucleotides downstream of the annotated start codon of the first gene. Although the classical view of transcription assumes that TSSs ordinarily occur upstream of operons, the presence of promoters and TSSs within some operons is accepted [57]. There are numerous reports of alternative and/or internal TSSs for well characterized operons (some examples include:[58]–[60]) and more recently RNA-Seq experiments have revealed the existence of internal promoters/TSSs on a global scale [9], [11], [14], [15], [22], [56]. For DC3000, the presence of internal promoters or TSSs has not been thoroughly investigated. Our study on HrpL-regulated genes [18] reports the presence of bioinformatically identified HrpL promoters internal to putative operons, although to date no experimental evidence for transcription initiation from those putative promoters has been provided. One example is a candidate HrpL-dependent promoter embedded in PSPTO_1399 (*hrpO*); our data provides evidence for transcription beginning immediately downstream of this internally located motif (in the E12+top data set), as well as evidence for several other internally located HrpL-dependent promoters. Our data also provides evidence for the presence of internal TSSs within several other predicted operons for DC3000. VIMSS predicts that PSPTO_1390 (*hrpT*) and PSPTO_1391 (*hrpV*) are co-transcribed, although our data suggests that *hrpV* may also be transcribed independently. Taken together, our data show that internal TSSs occur frequently in DC3000.

Promoter arrangement can lead to transcriptional interference [61], [62]. We identified over 100 cases in which promoters were located within 100 bases of one another (regardless of strand) and in 17 of these cases the promoter motifs actually overlap (by at least 1 bp). Since our analysis focuses only on TSSs for which we can identify an accompanying promoter motif, it provides a low-end estimate for promoters positioned in these ways Although we did not investigate these situations more closely, neighboring promoters may interfere with each other if both are active due to constraints imposed by polymerase size, collisions due to transcription on different strands, or complementarity between transcripts [61], [62].

In eukaryotes, most promoters are now thought to have multiple, closely spaced initiation sites (TSS clusters or transcriptional start site distributions) instead of single initiation points. It is not yet clear whether individual promoters exhibit these patterns or if the observed patterns are an averaged view of less complex initiation events across different subpopulations of cells. In bacteria the presence of transcription start clusters is less well described on a large scale. There are several examples of individual genes that have been examined in detail that have been shown to have multiple TSSs within a few nucleotides of one another. Others have reported multiple TSSs per promoter region [9]. More recently, RNA-Seq has detected very closely clustered TSSs for some transcripts in *Geobacter* [11] and the archaeal organism *Sulfolobus solfataricus* [22]. Closer examination of our 5′-end capture data often reveals what appears to be TSS clustering directly downstream of promoter motifs. For example, a majority of the HrpL and PvdS-dependent promoters have multiple, closely-spaced TSSs associated with them. The clusters are reproducible between biological replicates and the counts at each site follow the start site nucleotide preferences demonstrated in E. coli (A>G>U>C) [12], [25], [47]. We do not know the significance of this observation, but the selection of an initiating nucleotide may provide subtle opportunities for regulation in terms of transcript abundance (depending on available nucleotide pools) or influence the potential for alternative secondary structures [25], [63], [64].

The large amount of data recently collected regarding bacterial transcriptomes has changed our perception of the processes by which transcription occurs in bacteria. The discovery of alternative transcripts within operons, multiple promoters and TSSs per gene, antisense activity, and internal promoters within operons on a global scale has revealed immense complexity that we do not fully understand and furthermore has questioned the classical definition of operons [57]. It will be particularly interesting to determine how these processes are used to regulate pathogenesis in *P. syringae* and other bacterial pathogens.

## Supporting Information

Figure S1 **Unsaturated plots.** (a). Unsaturated scatter plot of TAP-only treated biological replicate 1 (E1) compared to TAP-only treated biological replicated 2 (E2). (b) Unsaturated scatter plot of Exonuclease treated biological replicate 1 (E1+) compared to exonuclease treated biological replicate 2 (E2+). (c) Unsaturated scatter plot of Exonuclease treated combined samples (E12+) and TAP-only combined samples (E12−).
(TIF)

Figure S2 **A histogram of the ratios of RNA-Seq read counts downstream and upstream of TSS's.** These ratios were computed by first counting the number of reads in the RNA-Seq sinister profile, 10 bp upstream and 10 bp downstream of the 5′-end of all 1827merged E12+Top peaks. Then, for each peak, we compute the ratio: log10(1+downstream_count) log10(1+upstream_count). The mean of the ratios was 0.40 (std 0.64).
(TIF)

Figure S3 **A histogram of the log_10 of the distances from TSS's to start codons.** To compute these distances, we first selected the set of E12+Top peaks that fell within 1000 bps upstream of annotated CDSs. This set included 1552 of the 2510 TSSs. The mean length of these 5′UTR regions was 77.8 bps.
(TIF)

Table S1 **Summary of 5′-end RNA-Seq data for** ***P. syringae*** **DC3000.**
(XLSX)

Table S2 **Distribution of unique positions.**
(XLS)

Table S3 **Transcriptional start sites confirmed by 5′RACE.**
(XLS)

Table S4 **Transcriptional start sites for putative RpoF regulated genes.**
(XLSX)

Dataset S1 **A profile for the DC3000 chromosome that can be loaded into Artemis to visualize the sequencing results of the Tap only treated sample E1**−**.**
(RAR)

Dataset S2 **A profile for the DC3000 chromosome that can be loaded into Artemis to visualize the sequencing results of the Tap only treated sample E2−.**
(RAR)

Dataset S3 **A profile for the DC3000 chromosome that can be loaded into Artemis to visualize the sequencing results of the exonuclease and Tap treated sample E1+.**
(RAR)

Dataset S4 **A profile for the DC3000 chromosome that can be loaded into Artemis to visualize the sequencing results of the exonuclease and Tap treated sample E2+.**
(RAR)

Dataset S5 **A merged profile for the DC3000 chromosome that can be loaded into Artemis to visualize the sequencing results of biological replicates E1− and E2−.**
(RAR)

Dataset S6 **A merged profile for the DC3000 chromosome that can be loaded into Artemis to visualize the sequencing results of biological replicates E1+ and E2+.**
(RAR)

Dataset S7 **A profile for the DC3000 chromosome containing the classification of each genomic location.**
(RAR)

Dataset S8 **Python script, make-class-scatter.py, for the three-way classification of 5′-ends.**
(RAR)

Dataset S9 **gff file of E12+Top peaks.**
(RAR)

Dataset S10 **gff file containing the resulting ranges for 40 bps upstream of E12+Top peaks.**
(RAR)

Dataset S11 **Data file containing the FASTA file of 40 bps upstream of E12+Top peaks.**
(RAR)

Dataset S12 **MEME results.**
(RAR)
