# Supplementary figures and images for: Genome-Wide Identification of Transcriptional Start Sites in the Plant Pathogen Pseudomonas syringae pv. tomato str. DC3000
Source: PLoS One. 2011 Dec 28;6(12):e29335. doi: 10.1371/journal.pone.0029335 (PMC3247240; doi:10.1371/journal.pone.0029335)

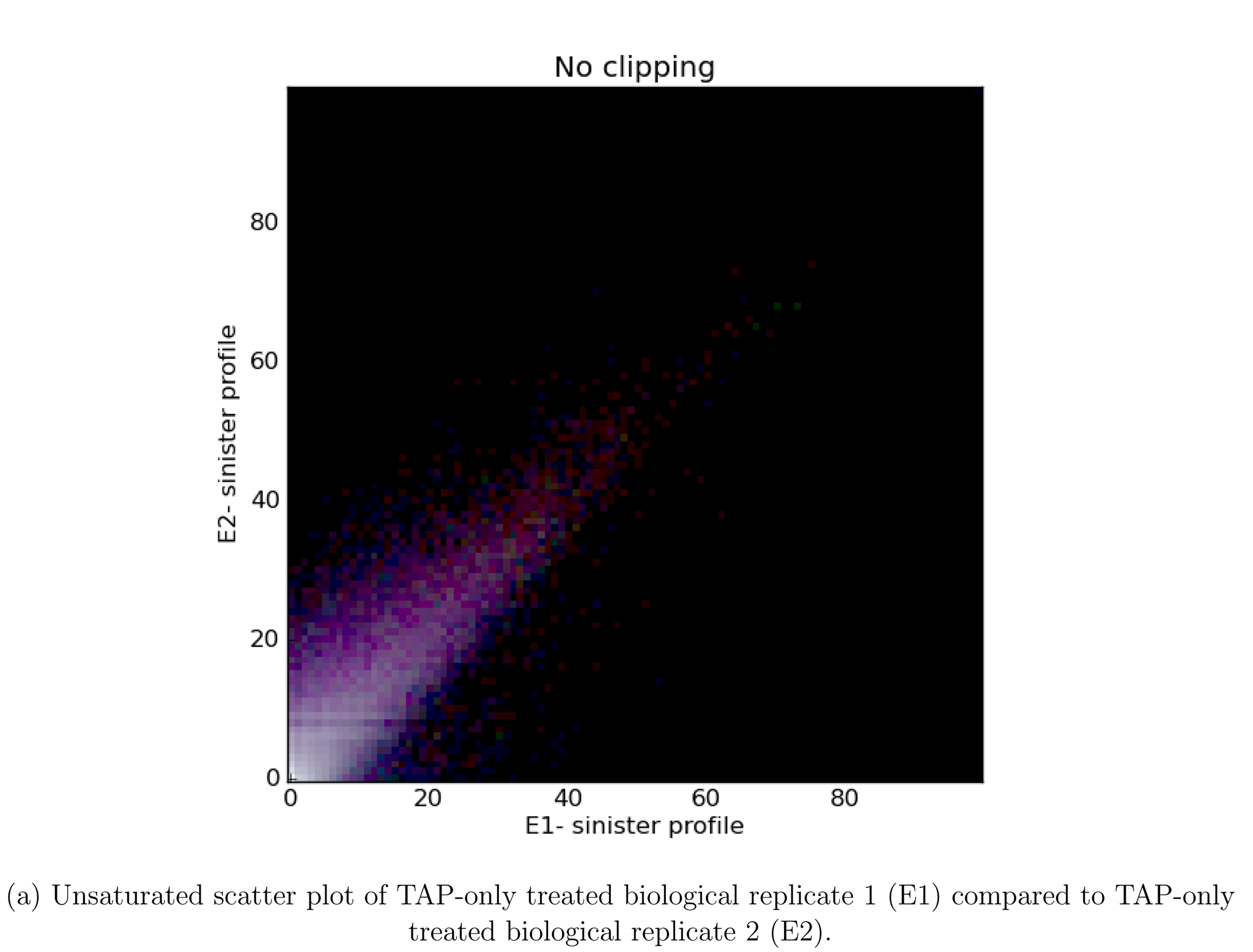

Supplement: Figure S1 — Unsaturated plots. (a). Unsaturated scatter plot of TAP-only treated biological replicate 1 (E1) compared to TAP-only treated biological replicated 2 (E2). (b) Unsaturated scatter plot of Exonuclease treated biological replicate 1 (E1+) compared to exonuclease treated biological replicate 2 (E2+). (c) Unsaturated scatter plot of Exonuclease treated combined samples (E12+) and TAP-only combined samples (E12−). (TIF) [file pone.0029335.s001.tif]

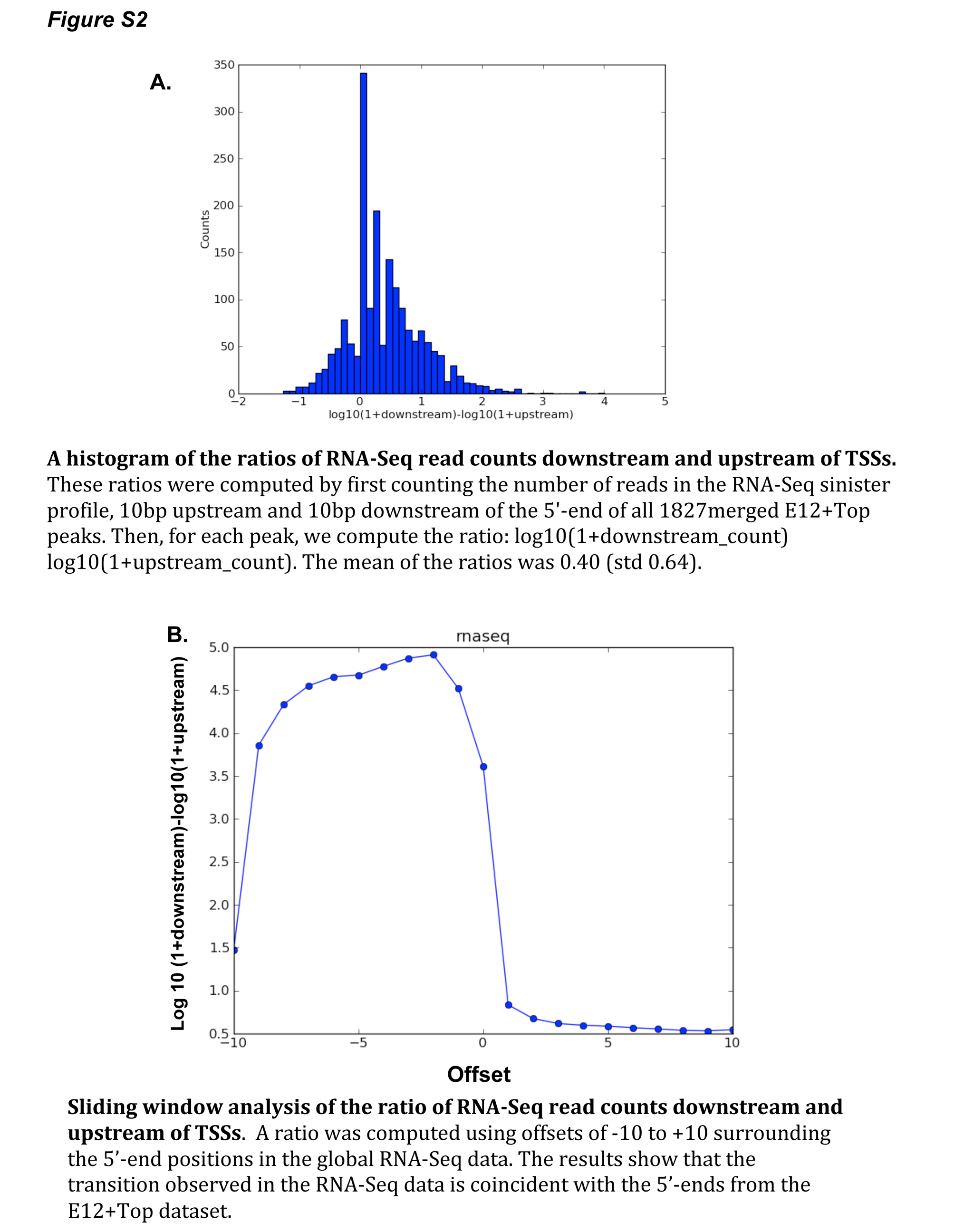

Supplement: Figure S2 — A histogram of the ratios of RNA-Seq read counts downstream and upstream of TSS's. These ratios were computed by first counting the number of reads in the RNA-Seq sinister profile, 10 bp upstream and 10 bp downstream of the 5′-end of all 1827merged E12+Top peaks. Then, for each peak, we compute the ratio: log10(1+downstream_count) log10(1+upstream_count). The mean of the ratios was 0.40 (std 0.64). (TIF) [file pone.0029335.s002.tif]

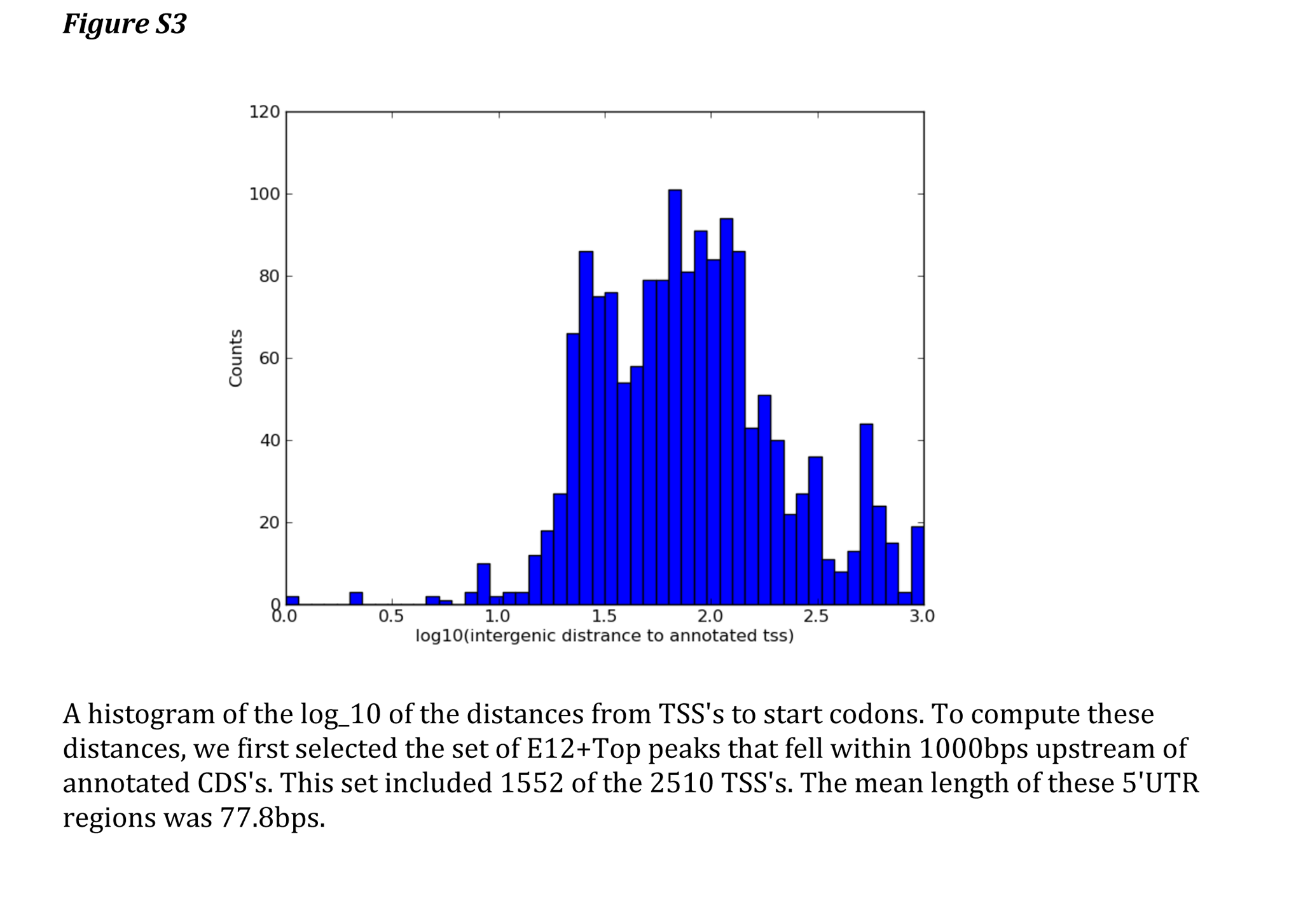

Supplement: Figure S3 — A histogram of the log_10 of the distances from TSS's to start codons. To compute these distances, we first selected the set of E12+Top peaks that fell within 1000 bps upstream of annotated CDSs. This set included 1552 of the 2510 TSSs. The mean length of these 5′UTR regions was 77.8 bps. (TIF) [file pone.0029335.s003.tif]
